# 

**Published:** 1870-09

**Authors:** 


*CONTENTS:*

ORIGINAL COMMUNICATIONS:
- ART. I. Uterine Fibroid of the Posterior Wall Successfully Removed. By A. Reeves Jackson, M.D....... 513
- ART. II. A Statistical Report of Diseases of the Ear. By E. L. Holmes, M.D................................ 521
- ART. III. Hydrate of Chloral. By James C. Bascom, M.D........... 524

ORIGINAL TRANSLATIONS:
- ART. I. Experimental Physiology. Experimental Investigations relative to the Directions of Rotary Movements due to Unilateral Encephalic Lesions. By Dr. J. L. Prevost, (continued).................. 525

SELECTIONS:
- Upon the Therapeutical Value of the Sulphites in Phlegmonous Angina. Paper read before the Sacramento Society for Medical Improvement. By G. G. Tyrrell ................. 539

EDITOR'S BOOK TABLE.............. 549

PAMPHLETS ........ .................. 551

EDITORIAL.............................. 560
- The Apostolic Succession. To Correspondents. A Modern Miracle. Dr. A. McFarland. Original Communications.

NEWS AND GOSSIP ..................... 564

LOOT ..................................... 568

MEDICAL SOCIETY PROCEEDINGS ... ... 578

